# Study on the influence of scaffold morphology and structure on osteogenic performance

**DOI:** 10.3389/fbioe.2023.1127162

**Published:** 2023-03-27

**Authors:** Jingyu Zhou, Shilang Xiong, Min Liu, Hao Yang, Peng Wei, Feng Yi, Min Ouyang, Hanrui Xi, Zhisheng Long, Yayun Liu, Jingtang Li, Linghua Ding, Long Xiong

**Affiliations:** ^1^ Department of Orthopedics, The Second Affiliated Hospital of Nanchang University, Nanchang, Jiangxi, China; ^2^ Institute of Clinical Medicine, Jiangxi Provincial People’s Hospital, The First Affiliated Hospital of Nanchang Medical College, Nanchang, Jiangxi, China; ^3^ The Second Clinical Medical College of Nanchang University, Nanchang, Jiangxi, China; ^4^ Department of Orthopedics, The First Affiliated Hospital of Nanchang University, Nanchang, Jiangxi, China; ^5^ Department of Orthopedics, Jiangxi Provincial People’s Hospital, The First Affiliated Hospital of Nanchang Medical College, Nanchang, Jiangxi, China; ^6^ Department of Traumatology, Jiangxi Provincial People’s Hospital, The First Affiliated Hospital of Nanchang Medical College, Nanchang, Jiangxi, China; ^7^ Department of Orthopedics, Jinhua People’s Hospital, Jinhua, Zhejiang, China

**Keywords:** scaffold, nanotechnology, hierarchically structured scaffold, porosity, bone tissue regeneration

## Abstract

The number of patients with bone defects caused by various bone diseases is increasing yearly in the aging population, and people are paying increasing attention to bone tissue engineering research. Currently, the application of bone tissue engineering mainly focuses on promoting fracture healing by carrying cytokines. However, cytokines implanted into the body easily cause an immune response, and the cost is high; therefore, the clinical treatment effect is not outstanding. In recent years, some scholars have proposed the concept of tissue-induced biomaterials that can induce bone regeneration through a scaffold structure without adding cytokines. By optimizing the scaffold structure, the performance of tissue-engineered bone scaffolds is improved and the osteogenesis effect is promoted, which provides ideas for the design and improvement of tissue-engineered bones in the future. In this study, the current understanding of the bone tissue structure is summarized through the discussion of current bone tissue engineering, and the current research on micro-nano bionic structure scaffolds and their osteogenesis mechanism is analyzed and discussed.

## 1 Introduction

With the aging population, the number of patients with bone defects caused by various bone diseases is increasing annually (El-Rashidy et al., 2017). Among them, large-scale bone defects are the biggest problem faced by orthopedic surgeons, which often require multiple operations, and the clinical treatment effect is poor, leading to delayed union or non-union, and even amputation. Presently, the gold standard for the clinical treatment of bone defects is pedicled autologous bone flap transplantation; however, the source of the autologous bone is limited, which increases the risk of wound infection, causes secondary injury to patients, and aggravates their pain. Biological factors, such as adding the vascular endothelial growth factor and bone morphogenetic protein into scaffolds, can regulate the directional differentiation of mesenchymal stem cells into vascular endothelial cells and promote bone regeneration (Won et al., 2020); however, the clinical effect is not good, the action is limited, the osteogenic effect is not ideal, and there is the possibility of inducing tumors (Silva et al., 2020; Hanusek et al., 2022). Cell or gene treatment methods have limitations such as being time-consuming, expensive, difficult to master in clinical applications, and potentially carcinogenic. The repair of massive bone defects is a clinical challenge in modern medicine, hence there is an urgent need to find a safe, convenient, and efficient means to promote bone regeneration.

In recent years, biomaterial scientists, represented by Academician Xingdong Zhang of Sichuan University, proposed the concepts of “tissue-induced biomaterials” and “*in vivo* tissue engineering” (Xing et al., 2019; Zhou et al., 2021). In other words, the microstructure design of the material is carried out to endow the material with the ability to induce tissue regeneration (Xing et al., 2019). This promotes fracture healing without the addition of growth factors. This theory suggests a new method to guide the research and development of bone regeneration materials in the future. Additionally, by improving the internal structure of the bone scaffold, the performance of the scaffold can be further optimized. by adjusting its surface morphology, which can adjust the fate of the cells and promote the progression of osteogenesis. Therefore, through a reasonable design of the scaffold, the maximum therapeutic effect of the scaffold, promotion of healing of bone defects, and alleviation of pain in patients can be achieved.

In this study, structure influencing factors influence its characteristics, the current micro-nano structure scaffold design, and the structure influence mechanism of osteogenesis. The purpose of this study was to determine the influence of the scaffold structure on the scaffold performance and cell fate.

## 2 Bone structure

**FIGURE 1:**
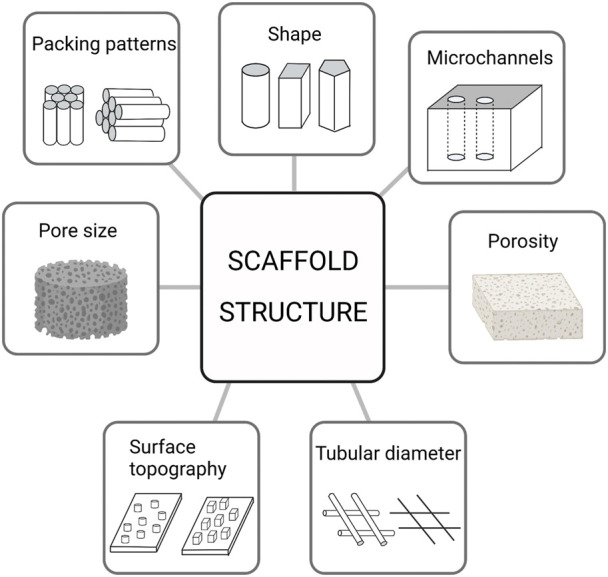
The primary structure of the scaffold at present.

Bone tissue is a natural nanocomposite material that is mainly composed of bone cells and a matrix around the bone cells. Bone cells are deeply embedded in a mineralized matrix, which senses mechanical stimulation and converts it into biological signals, regulates mineral homeostasis, promotes hematopoiesis and regulates secretion (Divieti Pajevic, 2013). The matrix components around the bone cells are mainly composed of organic and inorganic compounds. The main component of inorganic matter is calcium phosphate, which exists in the form of nano-hydroxyapatite crystals (Weiner et al., 1999); 90% of organic components are mainly type I collagen, and the rest are composed of lipids, growth factors, osteopontin, proteoglycan, adhesion proteins, and other molecules (Carvalho et al., 2018). Macroscopically, the bone tissue is composed of cortical and cancellous bones. Cancellous bones are mainly composed of trabeculae of different sizes, forming a high-porosity structure (up to 30%–90%) and a low elastic modulus. Cortical bones are mainly composed of a Haval bone plate, an inner interosseous plate, and an outer ring bone plate, with low porosity (5%–30%). The special structure of the bone tissue determines its special function; the high porosity of cancellous bone ensures the exchange of intramedullary nutrients and participates in the main metabolic process of bone tissue (Lin et al., 2016). The cortical bone plate is mainly composed of mineralized accumulation and precipitation of inorganic components, and the cortical bone has a high elastic modulus, high hardness, and low toughness, which play a major supporting role (Han et al., 2018). When the scaffold is implanted into a bone defect site, it mainly replaces the bone, temporarily supports the structure, and participates in the metabolism of the bone tissue. Therefore, scaffolds must have hierarchical structures and characteristics similar to those of the bone tissue. The hierarchical structure of scaffolds mainly includes macroscopic features such as the tubular diameter, shape, pore, and microchannels, and nano-microscopic features such as surface morphology and nano-pores (Figure 1). Its characteristics include inductivity, electrical conductivity, mechanical properties, hydrophilicity, hydrophobicity, cell compatibility, biodegradability, and biocompatibility. It is the key to the scaffold design for bone tissue engineering to adjust the scaffold structure and improve the scaffold characteristics (Giannitelli et al., 2014). Table 1 provides an overview about the influence of scaffold structure on scaffold characteristics.

**TABLE 1:** Influence of scaffold structure on scaffold characteristics.

Characteristic	Structure	Ref
Conductivity and inductivity	Surface characteristics	Graziano et al. (2007), Graziano et al. (2008)
	Pore size	Wang et al. (2013a), Zhang et al. (2022b)
Mechanical properties	Pore architectures	Lee et al. (2012)
	Stacking direction	Lee et al. (2012), Feng et al. (2017)
	Geometric shape	Zhao et al. (2019)
	Porosity	Boccaccio et al. (2016), Xiao et al. (2016)
Hydrophilicity and hydrophobicity	Atomic topology of Surface	Yu et al. (2018)
	Surface topography	Gagner et al. (2012), Wu et al. (2022)
	Tubular diameter	Gongadze et al. (2013)
Pore size and porosity	Pore size and porosity	Ma et al. (2000), Rnjak-Kovacina et al. (2011), Zhang et al. (2022a), Song et al. (2022)
Biodegradability	Cylindrical structure	Chew et al. (2016)
	Porosity	Zhang et al. (2019)
	Pore size	Kim et al. (2016)
Biocompatibility	Porosity	Dezfuli et al. (2012)
	Porosity distribution	Hamilton et al. (2009)

## 3 Influence of structure on scaffold characteristics

### 3.1 Conductivity and inductivity of scaffolds

The scaffold, which has a certain conductivity, must provide a growth surface for osteoblasts from the periphery of the implant bed and directional osteoblasts in the bone marrow. The pore diameter between 0.1 mm and 0.5 mm is regarded as the best distance for bone conduction, which may be related to the proliferation of pre-osteoblasts and better initial adhesion of osteoblasts (Hollister, 2005; Murphy et al., 2010). The induction of scaffolds means that biomaterials directly induce peripheral mesenchymal stem cells to differentiate into bone precursor cells and osteoblasts and further form bone tissue. Presently, most artificial bone products used in clinics only have bone conductivity, poor osteo-inductivity, and weak osteogenesis, and it is still difficult to solve large bone defects clinically (Ho-Shui-Ling et al., 2018). Numerous studies have reported that the conductivity and inductivity of scaffolds are affected by their structures (Humbert et al., 2019). The surface characteristics of the scaffold are related to osteogenesis, and a concave surface of the scaffold is more conducive to osteogenesis than a convex one (Graziano et al., 2008). The cells on the microcavity-rich scaffold released a significant amount of *BMP-2* and *VEGF* into the culture medium and expressed higher alkaline phosphatase activity, which induced bone tissue formation (Graziano et al., 2007; Wang H. et al., 2013) deduced that the macro-porous structure of the HA stent is beneficial for angiogenesis and osteo-induction. Macro-porous structures ensure nutrient and metabolic waste transport, vascular ingrowth, and direct osteogenesis (Murphy et al., 2010; Wang et al., 2021). This type of pore has an optimum size. In the research by Zhang et al. (2022b), it was deduced that *in vivo* experiments, the porous structure with a size of 400 μm is more conducive to ectopic bone growth, whereas *in situ* bone defects, the porous structure with a size of 600 μm has the largest area of new bone tissue. Yamasaki and Sakai (1992) emphasized that the existence of interconnected microporous structures (2–10 μm) can endow scaffolds with osteo-inductive characteristics; inward bone growth was not observed in similar materials with a dense morphology. On one hand, micropores can provide niches for cells that preferentially undergo osteogenic differentiation, and adsorb cells to settle in micropores by capillary forces (Polak et al., 2013). On the other hand, the microporous structure can increase the surface area of scaffolds and provides more adsorption sites for proteins or cells.

### 3.2 Mechanical properties of scaffolds

When the scaffold is implanted into the bone defect site, it should meet certain mechanical properties, provide support for the fracture end, and simultaneously, should be similar to the mechanical properties of human bone tissue. Otherwise, it would cause stress concentration and fracture recurrence. The mechanical properties of the scaffold are closely related to the structure, whereas those of the same material can be changed by changing the internal structure. Wang et al. (Feng et al., 2017) used a three-dimensional (3D) printing system to prepare three types of biomimetic scaffolds with different packing patterns (i.e., cross-packing, quartet close-packing, and hexagonal close-packing patterns). Among them, the compressive strength of the hexagonal dense stacked bionic scaffold was the highest (the range was 30–46 MPa). Furthermore, the mechanical properties of the scaffolds can be enhanced by controlling the pore architecture and stacking direction. Among the scaffolds made of PCL/PLGA blends, the highest compressive strength of the triangular scaffolds is 9.81 Mpa, which can be used to enhance their mechanical properties, whereas the compressive strength of lattice and staggered scaffolds is 6.05 Mpa and 7.43 Mpa, respectively (Lee et al., 2012). Presently, the influence of the differences in the construction direction, material structure, and geometric shape of the support on stress, can be determined using the finite element model to further design the support and improve its performance (Boccaccio et al., 2016; Zhao et al., 2019; Xiao et al., 2016) used finite element modelling (FEM) to redesign the scaffold microstructure and improve its bending strength without significantly lowering its compressive strength and ability of bone regeneration *in vivo*. The data verified the prediction of the finite-element simulation. This scaffold, with a different pore gradient structure, composed of a less porous outer region and a more porous inner region, exhibited a flexural strength (34 ± 5 Mpa) that was more than twice the value of the uniform grid-like microstructure (15 ± 5 Mpa) and a higher compressive strength (88 ± 20 Mpa) than the grid-like microstructure (72 ± 10 Mpa). It can better imitate the microstructure of human long bones and provide a more reliable guarantee of bone repair.

### 3.3 Hydrophilicity and hydrophobicity

The hydrophilicity and hydrophobicity of the material surface affect the cell morphology and surface adhesion level. Cells can spread, proliferate, and differentiate on hydrophilic surfaces, whereas hydrophobic surfaces adsorb more proteins. The hydrophilicity and hydrophobicity of the scaffold surface are related to its topological structure, and Yu et al. (2018) showed that silica exhibits a hydrophilic-to-hydrophobic transition driven by its silanol surface density. The topological constraint theory was applied to show that the surface reactivity and hydrophilic/hydrophobic character of silica are regulated by the atomic topology of its surface. The surface structure of the scaffold can affect the hydrophilicity and hydrophobicity of the scaffold, and further, affect protein adsorption. Gagner et al. (2012) used wet chemical methods to synthesize gold nano-cubes (AuNC) with 100 facets and gold nano-ctahedra (AuNO) with 111 facets. Their chemical compositions are similar, but their protein adsorption level is different. When the protein concentration was saturated, the protein was adsorbed on AuNO with a higher surface density. The different surface structures may affect the packing density of the negatively charged ligands and further affect their affinity for protein adsorption. Wu et al. (2022) formed a nano-rod coating on a surface of the scaffold using a hydrothermal method, which improved the hydrophilicity of the scaffold. In contrast, nano-rod coating significantly increases biological activity. Moreover, by changing the internal tubular diameter of the scaffold, hydrophilicity and hydrophobicity can also be affected, thus affecting the protein adsorption level. Gongadze et al. (2013) quantitatively detected fibronectin content by ELISA and found that the adsorption of fibronectin on the surface of TiO_2_ nanotubes with different diameters was quite different.

### 3.4 Pore size and porosity

Porosity refers to the ratio of the pore volume to the total volume of materials, which is a morphological property independent of materials (Karageorgiou and Kaplan, 2005). Natural bone, as a gradient porous structure, has a complex structure and can meet expected physiological functions. The cancellous bone is mainly composed of trabeculae with a high porosity of 50%–90%, whereas the cortical bone has only 5%–10% porosity. The pore structure is essential for cell nutrition, proliferation, migration, tissue vascularization, and new tissue formation (Salerno et al., 2012). Generally, larger pores are conducive to blood vessel growth and abundant material exchange, which are more suitable for cell survival (Artel et al., 2011). For scaffolds with pore sizes between 250 and 500 μm, chondrocytes show preferential proliferation and ECM production (Lien et al., 2009). The pore structure facilitates cell adsorption and provides anoxic conditions that induce osteochondral formation before osteogenesis (Karageorgiou and Kaplan, 2005). However, the ability of larger pores to promote cell infiltration has been proven to override the beneficial effect of a larger initial cell attachment surface area provided by smaller pores (Loh and Choong, 2013). Presently, it is generally accepted that scaffolds with 300–800 μm through macropores and secondary capillary micropores (≤10 μm) inside the macropores show good osteo-inductivity (Li T.-T. et al., 2021). The porosity and pore size of a scaffold directly affect its function in biomedical applications. Porosity is proportional to the surface area, and the surface area of the scaffold material gradually increases with an increase in the porosity, which may help to transport nutrients and oxygen or make more cells grow inward; cells are more likely to adhere to the surface of the scaffold material (Cychosz et al., 2017). However, owing to the large void volume, compressive strength are reduced, and the degradation process of the scaffold is promoted (Karageorgiou and Kaplan, 2005). Additionally, the porosity and pore size affect cell proliferation and differentiation. Ma et al. (2000) constructed high porosity (HP, 89.6%, average pore size 39 μm) and low porosity (LP, 84.9%, average pore size 30 μm) using polyethylene terephthalate by hot compression technology. The proliferation rate of ED27 cells in the LP co-culture system was higher than that in the HP co-culture system, but the differentiation activity of the ED27 cells in the HP co-culture system was higher than that in the LP matrix, which may be related to the small pores in LP, limiting the cell cluster and affected cell differentiation. Additionally, the porosity and pore size can affect the ECM composition of the extracellular matrix. Fibronectin and type I collagen were deposited in fibroblasts cultured in a synthetic human elastin scaffold with high porosity and a large average pore size during cell culture, and the expression of the collagen-related marker genes was also up-regulated (Rnjak-Kovacina et al., 2011).

### 3.5 Biodegradability

Biomaterials should have the ability to degrade with time *in vivo* so that new tissues can grow and replace old ones, to increase the growth space for new tissues, and finally to make new bone tissues completely replace scaffolds and restore the normal physiological functions of bone defects (Yang et al., 2019). The tissue growth rate is different in different parts, For example, the lower limb requires negative weight, the fracture stabilization takes time, and material degradation time can be delayed; head and face or upper limb fracture stabilization time is relatively short, the material degradation rate can be accelerated, and the ideal biomaterial has controlled rate degradation according to the tissue growth rate. The scaffold structure is closely related to its degradability. Chew et al. (2016) prepared PLGA scaffolds with different structures to evaluate the influence of structures on scaffold degradation and found that the degradation ability of the thin strand scaffolds, which had the highest SVR, was stronger than that of the coarse and fine chain structures because the increase in the surface area allows more contact between water molecules and degradable ester groups in the polymer. Zhang et al. (2019) prepared Ga-P scaffolds with different pores using 3D printing technology. The porosity increased non-linearly with an increase in the pore size, and the degradation rate of the scaffolds also increased. Kim et al. (2016) prepared magnesium phosphate ceramic scaffolds containing macropores (100 µm) but micropores of different sizes by combining 3D printing with salt immersion. Compared to scaffolds without micropores, scaffolds containing micropores exhibited faster biodegradation. Therefore, by improving the scaffold structure, individual schemes can be formulated to satisfy different degradation requirements.

### 3.6 Biocompatibility

Biocompatibility refers to the properties of living tissues that react with inactive materials (Crawford et al., 2021). Any implant in the body causes rejection. Currently, the purpose of the scaffold design is to regenerate tissues and support cell activity without causing toxic side effects or host reactions (Hussein et al., 2016). Therefore, in the design and application of stents, *in vivo* rejection must be minimized. Presently, the most common strategy is to increase the biocompatibility of scaffolds by combining them with natural materials. It is generally believed that scaffolds constructed from natural materials such as hydroxyapatite, chitosan, and collagen (Akilbekova et al., 2018) have good biocompatibility (Cheburu et al., 2011; Park et al., 2019; Cursaru et al., 2022), but natural materials are difficult to process, unstable in material properties, and poor mechanical properties, and some materials such as collagen can have immunogenicity (Shahab et al., 2012). Additionally, the scaffold structure can affect biocompatibility. Dezfuli et al. (2012) deduced that the porosity distribution influences cell viability and proliferation. High porosity indicates a large surface area, and scaffold cells with a large surface area have high viability. In addition to the shape of the scaffold, the shape of the internal particles also affects cell viability (Hamilton et al., 2009; Zhao et al., 2013) co-cultured cells with nano-sized hydroxyapatite (nHA) of different shapes. They found that needle- and plate-shaped nHA resulted in the most significant cell death in BEAS-2B cultures compared to sphere- and rod-shaped nHA.

## 4 Structure of scaffold

### 4.1 Hierarchical structure of bone

**FIGURE 2:**
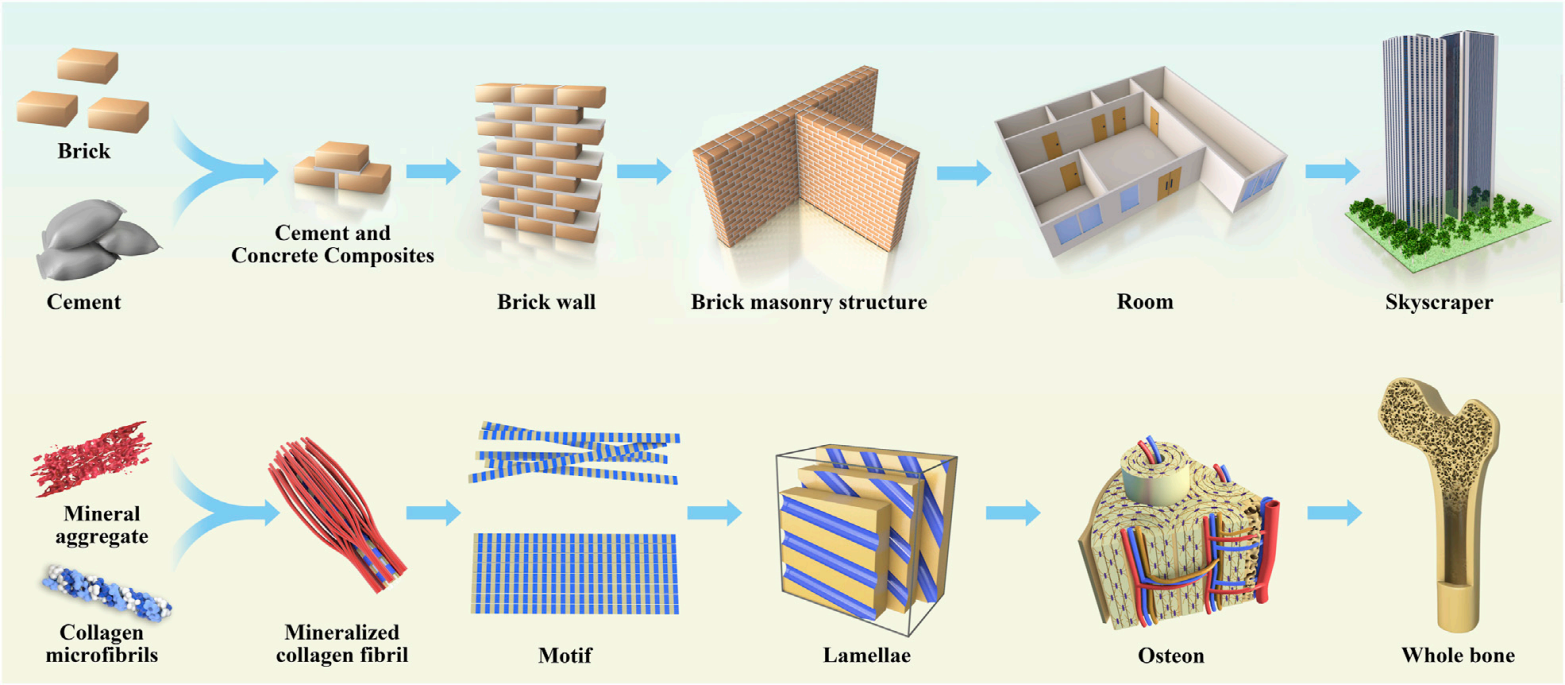
The anatomical structure of bone is analogical to architecture, which is divided according to hierarchy, and each hierarchy plays its own function and forms a whole together.

Natural bone is a non-homogeneous anisotropic nano-composite material whose main components are organized in layers into several structural levels ranging from macroscopic to nanoscale levels (Figure 2). The cognition of the hierarchical structure of bone tissue is a gradual process. Weiner et al. (1999) first proposed that the lamellar bone is composed of lamellar unit structures by measuring the angle deviation of the collagen fibers using an SEM microscope and proposed that the lamellar bone has seven hierarchical structures. In 2014, Reznikov et al. (2014) proposed a three-dimensional bone by the focused ion beam electron microscopy and serial surface observation method for further observation of the architecture and proposed further improvements to this theory by dividing the lamellar bone into nine structures. In 2018, the structure of mineralized collagen fibers was subdivided using STEM tomography, followed by the proposal in Science that the natural bone has a complex multilayered structure at different scales ranging from the millimeter level to the micro-nanometer level for a total of 12 levels (Reznikov et al., 2018). Natural bone contains a rich hierarchical structure that provides directions for scaffold construction and structural optimization. The construction of multi-level structural scaffolds from the macrocosm to the microcosmic bone by simulating the natural bone structure is a major challenge in current bone tissue engineering.

### 4.2 Hierarchical structure of the scaffold

The rich hierarchical structure is a typical feature of the natural bone structure, which not only provides excellent biological properties to materials but also provides an ideal microenvironment *in vivo*, which contains rich and diverse signal clues affecting the cell fate (Iacoviello et al., 2020). Current bone repair biomaterial scaffolds aim to reproduce such a microenvironment, promote inward cell growth and differentiation, and be applied in the vascularization of osteogenesis. Therefore, biomaterial scaffolds with porous nanostructures and 3D layered structures are the most promising bone substitutes for simulating natural bones (Vordemvenne et al., 2020; Luo et al., 2020) simulated the dimensions of fibers in human extracellular matrix (ECM) using the membrane-liquid interface culture method, to produce a novel bacterial cellulose/cellulose acetate scaffold, which exhibited an interpenetrated nano (42 nm) and submicron (820 nm) fibrous structure and contained nanopores and macropores. The novel scaffold exhibited enhanced cell proliferation, alkaline phosphatase activity, and gene and protein expression compared to single bacterial cellulose and cellulose acetate scaffolds. Thomas et al. (Vordemvenne et al., 2020) studied collagen sponges. They observed that the collagen sponge had 60.66 ± 24.48 μm pores and 32.97 ± 1.41 nm nano-pores, and coated it with SiO_2_ nanoparticles with a size of approximately 146 nm to cover up the original morphology and structure. Subsequently, the levels of bone growth and healing decreased in the skull defect model. Zhang et al. (Wu et al., 2020) fabricated biomimetic natural wood-like hierarchically structured scaffolds with first-level macropores (∼100–600 μm) and second-level micro/nanoscale pores (∼100–10,000 nm) by 3D printing technology. A micro/nano-whisker coating was prepared on the surface of the scaffold by hydrothermal treatment. This hierarchically structured scaffold exhibited excellent osteo-inductive activity. Li et al. (2019) inspired by the composition, structure, and function of hot dogs, printed hollow bioceramic tubes through improved 3D nozzles through 3D printing technology and bidirectional freezing technology and well-dispersed bioceramic slurry was placed in hollow ceramic tubes and fixed by bidirectional freezing technology. Finally, ice crystals were sublimated by the freeze-drying method. Finally, a hierarchical hot dog scaffold composed of a hollow tube structure embedded with a bioceramic rod and a uniformly arranged layered microstructure was successfully prepared. Compared to a non-hot dog-like system with the same chemical composition, this layered hot dog-like structure had a double-layer macro-porous and microporous structure, and its drug loading capacity and drug release time were significantly improved. The drug release time was 90 days. The scaffold has a large surface area, which is beneficial for cell adhesion and can promote the expression of osteogenic genes, such as *Runx2, OCN,* and *OPN*.

### 4.3 Macrostructure of scaffold

**FIGURE 3:**
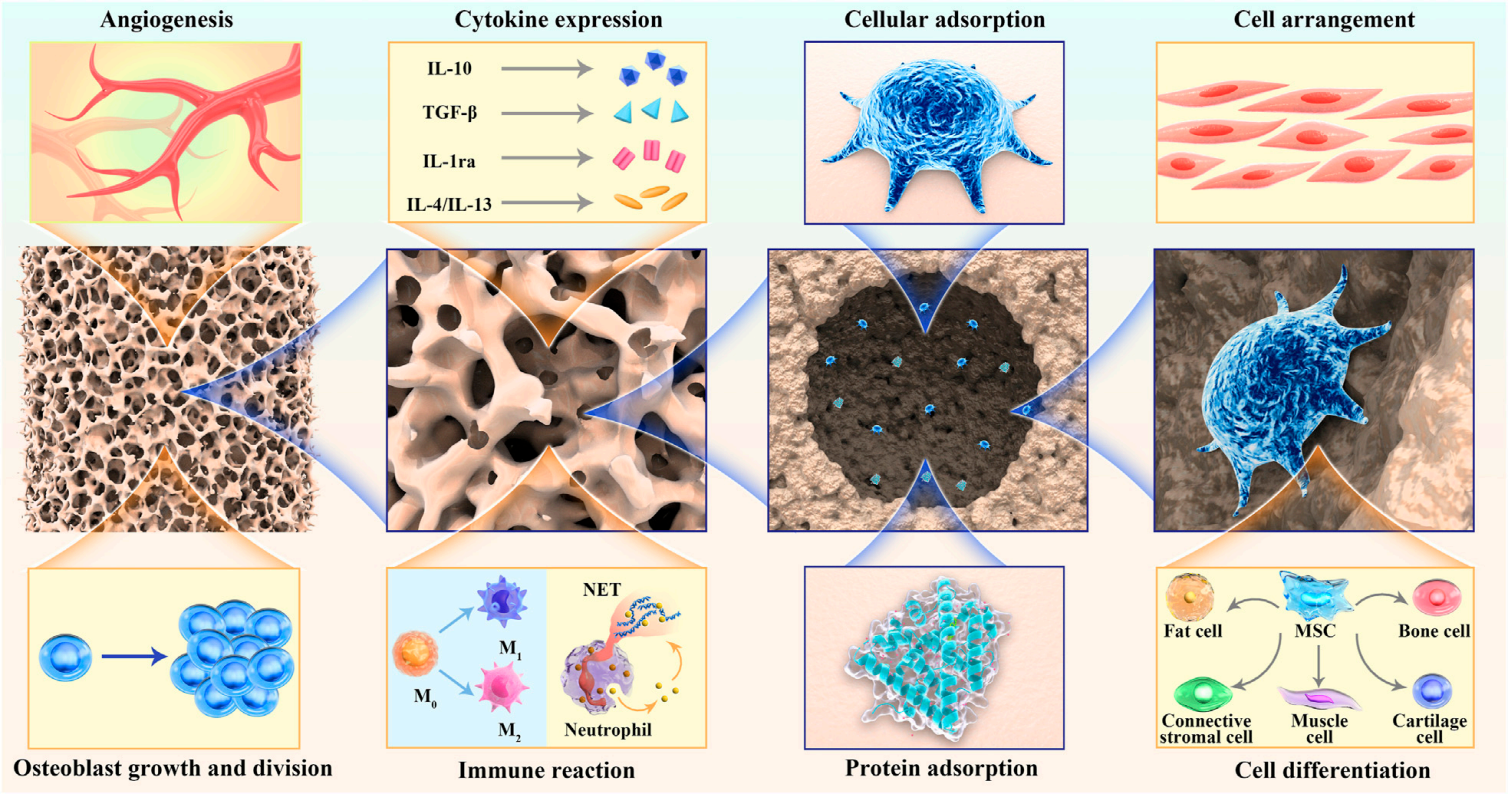
Scaffolds with different hierarchical structures play different functions.

Current studies have confirmed that different scales of hierarchical structures have different functions (Figure 3): small structures (<10 μm) are more easily impregnated by tissue fluid, creating more sites for cell adsorption (Perez and Mestres, 2016); medium structures (20–40 μm) help promote the conversion of primary macrophages to the M2 type and upregulate anti-inflammatory gene expression to suppress the host immune response to grafts (Sadtler et al., 2016), which facilitates the inward growth of host cells, especially MSCs; the large scale structures (>100 μm) facilitate angiogenesis, cell homing, and colonization, and provide a site for cell colony formation (Karageorgiou and Kaplan, 2005; Murphy et al., 2010; Zhang et al., 2018). Additionally, the size of the pore structure affects cell proliferation and differentiation. Adipose stem cells were inoculated onto PCL stents prepared with different pore sizes (100 μm, 200 μm, and 400 μm) and placed under chondrogenic differentiation conditions for 21 days. The results showed that for the 100 μm and 200 μm pore sizes, the ASC cells were evenly distributed and proliferated in higher numbers, whereas in the 400 μm pore size scaffolds, the cells tended to aggregate, and proteoglycan production and chondrogenic markers were significantly higher in the 400 μm pore size scaffolds than in the 100 μm and 200 μm pore sizes (Im et al., 2012). Although many 3D-printed biological scaffolds with high porosity have been prepared for tissue regeneration, the micropores in the scaffolds cannot form channel structures, which hinders the formation of the basic vascular system and internal new bone tissue (Yan et al., 2019; Liu et al., 2023). Adding microchannels to the scaffold can induce endothelial cells to form a basic vascular system, promote oxygen/nutrition perfusion, and induce tissues to grow inward along these channels (Rnjak-Kovacina et al., 2019; Wen et al., 2021). Feng et al. (2017) fabricated lotus-root-like biomimetic materials with parallel multichannel structures *via* a modified 3D printing strategy. Owing to the existence of microchannels, the porosity and specific surface area of this bionic structure material were obviously improved. Compared with traditional 3D printing materials, lotus root-like bionic materials have significantly improved the attachment and proliferation of BMSCs *in vitro* and osteogenesis, as well as angiogenesis *in vivo*.

### 4.4 Nano-microstructure of scaffold

The nanostructured composition of the bone tissue consists mainly of nano-hydroxyapatite and collagen fibers. The main component of hydroxyapatite is calcium phosphate crystals, which are mainly located inside collagen fibers. In contrast, collagen, as an endogenous structural protein, makes up the organic component of the bone tissue, formed mainly by the self-assembly of three amino acid peptide chains with 31.93 ± 14 nm pores on the surface (Greiner et al., 2019), providing an attachment surface for the hydroxyapatite crystals (Xu et al., 2020) and promoting their better mineralization (Nudelman et al., 2010). During bone formation, nHA crystals are mainly arranged along the c-axis parallel to the collagen fibers and organize the biomineralization along the fibers in a periodic, staggered fashion, and thus constitute the main nanostructure of the bone tissue (He et al., 1999). This kind of nano-microstructure structure mainly involves nano-scale, including surface morphology and nano-pores. Nanostructures are essential for tissue engineering, not only to modulate hydroxyapatite crystal mineralization but to increase the mechanical strength of the bone and further influence physical and chemical properties, such as the crystal polymorphism and melting point after crystal nucleation (Hamilton et al., 2012; Jiang and Ward, 2014) but also to guide cells to assemble and attach in a specific way or a specific area on the scaffold, ultimately affecting the fate of the cells (Singh et al., 2014). Some studies have shown that the preparation of nanotube structures with diameters ranging from 30 to 50 nm by mimicking the surface pores of collagen fibers can promote mineralization (Yang et al., 2014; Minagar et al., 2015; Zhang et al., 2017). In a study by Cantaert et al. (2013), it was observed that the pore size was closely related to the degree of crystal orientation, and the degree of crystal orientation at 50 nm was better than that at 200 nm. Moreover, numerous studies have shown that the nanosphere structure affects the biological properties (Manoukian et al., 2018). Zhen et al. deduced that nano-topology exhibits better cell adhesion and proliferation than micro-topology, thus increasing the biomechanical strength of implants (Geng et al., 2020b). Meanwhile, Xia and his research team (Xia et al., 2020) deduced that increasing the nanopore diameter inhibits the initial adhesion of BMSC cells, but can promote a larger diffusion area of cells and an increased expression level of ALP, osteopontin, osteocalcin, and type I collagen, which are more favorable for osteogenesis. Greiner et al. successfully constructed self-assembled silica nanoparticles by a thermally induced cross-linking reaction with oleic acid–silica nanocomposites with a pore size of approximately 34 ± 14 nm and demonstrated that the surface pore size of endogenous type I collagen fibers could promote stem cell osteogenic differentiation (Greiner et al., 2019). Moreover, surface nano-topography is sufficient to regulate cellular behavior. Park et al. prepared vertical titanium dioxide nanotubes with diameters of 15 nm and 100 nm, and MSCs grown on 15 nm diameter nanotubes exhibited increased expression of the bone morphogenetic protein-2, which promoted osteogenic differentiation, whereas the 100 nm diameter nanotubes exhibited reduced cell adhesion levels, increased apoptosis, and promoted chondrogenic differentiation (Oh et al., 2009). Dalby et al. prepared nanogroove structures of different depths using polymer layering and colloid lithography. Cytoskeleton staining of the HMSCs cell co-culture revealed that the cell spreading area increased and the expression of the stress fibers increased. Additionally, HMSC’s react strongly to surface features down to 10 nm in height with a low aspect ratio and enriched osteoblast differentiation (Dalby et al., 2006a).

In conclusion, the microstructure of the scaffold plays a different role depending on the surrounding bone tissue hierarchy, constructing bone regeneration scaffolds, further studying the biological properties of different layers of bone tissue, and providing a basis for subsequent studies on multilevel structural scaffolds. However, the effect of these scaffold structures on osteogenesis can be summarized as follows.

## 5 Mechanism of scaffold structure promoting osteogenesis

### 5.1 Biomechanics of material morphology

**FIGURE 4:**
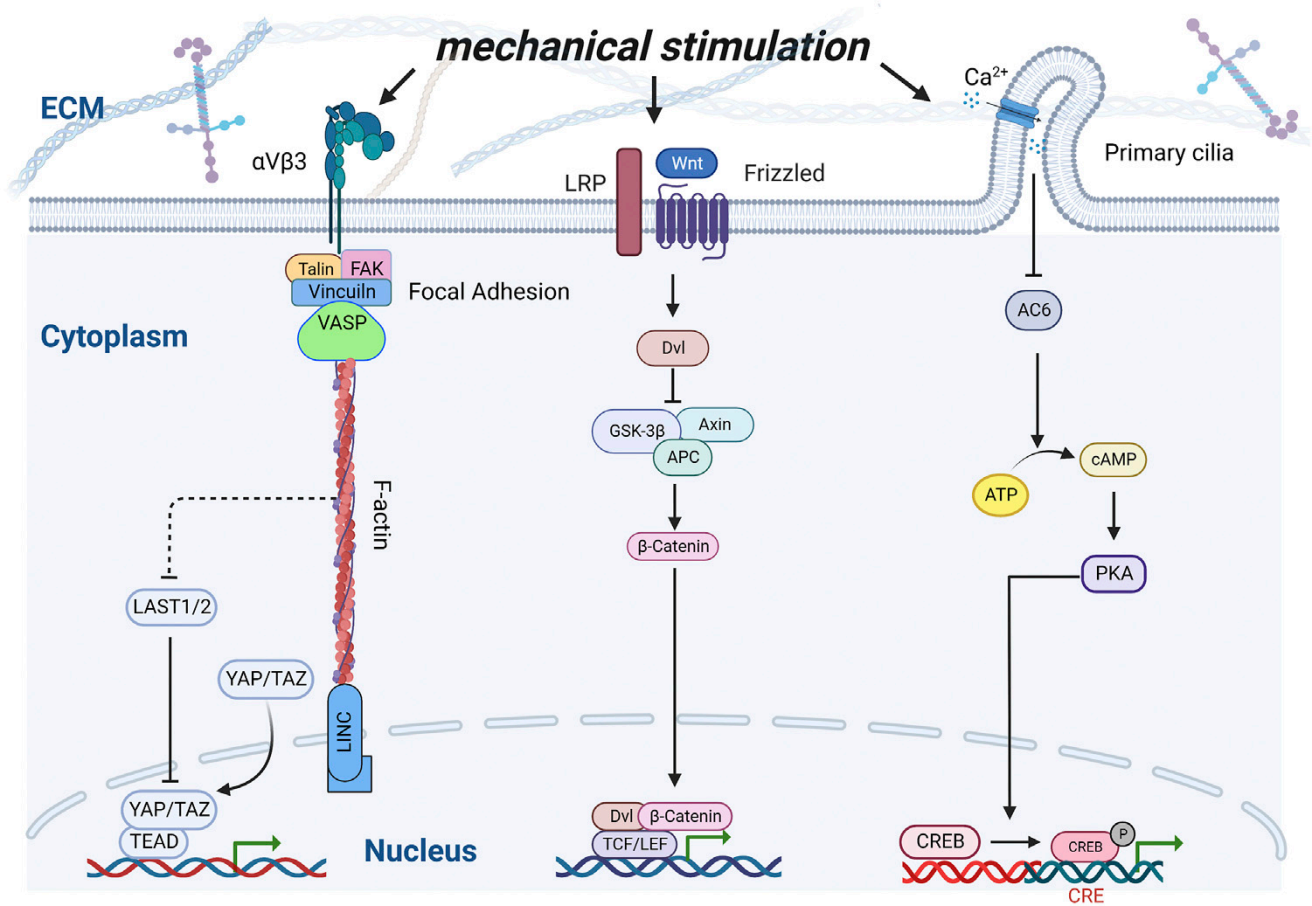
Schematic of interactions of various signaling pathways under mechanical stimulation. Integrins, Wnt receptors, and Ca2+ channels were stimulated by mechanical stimulation, thereby inducing a series of transcription factors to regulate osteoblast proliferation and differentiation.

The internal structure of a material can induce cell deformation and regulate gene expression (Liu and Ding, 2020). When the material is implanted into the body, the cells adhere to the surface of the material, and the material morphology induces cell deformation, causing changes in the cell surface pressure and internal tension, which are transmitted to the nucleus through a series of signals, which finally causes the cells to respond (Campbell and Humphries, 2011; Könnig et al., 2018). Therefore, extracellular matrix mechanical signals play a crucial role in the regulation of physiological processes, such as the maintenance of the cell behavior and function (Hannezo and Heisenberg, 2019). It is important for the development, growth, and maintenance of the bone. Numerous receptors (Nguyen and Jacobs, 2013; Bertrand et al., 2020), are distributed on the surface of bone cells and participate in cell mechanical transduction. Integrin-containing focal adhesions, *Wnt* receptors, including Lrp5, primary cilia, voltage-gated calcium channels, and connexin-based gap junctions are the major mechanisms implicated in bone cells (Li X. et al., 2021) (Figure 4). When a mechanical force acts on the cell membrane, it stimulates the autophosphorylation of the focal adhesion kinase (Michael et al., 2009), and further promotes the sliding of F-actin on myosin II, which causes the contraction of the cytoskeleton and finally transmits it to the linker of the nucleoskeleton and cytoskeleton complex, thus regulating the transport of the transcription factors (Wang et al., 2005; Speight et al., 2016). Zhen et al. affected the mineralization of the surface coating by adjusting the pH. Compared with the flake morphology prepared at low pH, the expression levels of ITG α5 and ITG β1 related to the cell adhesion level increased on the surface nano-needle strontium-substituted apatite coating prepared at high pH, and the expression of related osteogenesis related genes such as Runx2, ALP, Col-I, and OCN also increased significantly (Geng et al., 2021). Moreover, F-actin opposes the Yes-associated protein (*YAP*) and transcriptional coactivator with PDZ-binding motif (*TAZ*) phosphorylation through inhibition of the kinases *LATS1* and *LATS2* (Halder et al., 2012). As a transcriptional co-activator, *YAP/TAZ* can up-regulate the expression of the vascular endothelial growth factor, transforming growth factor-β (TGF-β), bone morphogenetic protein-2 (BMP-2), and other growth factors (Pefani et al., 2016; Azad et al., 2018; Sivaraj et al., 2020), and plays an important role in the fracture healing process (Zarka et al., 2021). In scaffolds, high curvature surfaces or small pores (<125 µm diameter) can up-regulate the phosphorylation of *YAP*-related proteins, whereas relatively low curvature or large pores (>250 µm diameter) can down-regulate the phosphorylation of *YAP* and increase its nuclear translocation, and transcriptional activation reverses osteogenic differentiation (Swanson et al., 2022). The mechanical stimulation of the *Wnt* pathway involves binding of the *Wnt* ligand to the transmembrane receptor *Fzd,* which forms a complex with *LRP5*. *Wnt-Fzd* binding causes *Dvl* to inhibit *Axin/APC/GSK-3β,* releasing β-catenin to the nucleus and binding to the *TCF/LEF* family as a coactivator of transcription (Bertrand et al., 2020), which can up-regulate the expression of osteoblast-related genes, such as *Col-1*, *ALP* and *OCN*, thus controlling the osteoblast differentiation and bone development (Li et al., 2018). Primary cilia are mechanically sensitive to flow and serve as part of the calcium signaling system (Saternos et al., 2020). Flow-induced calcium influx inhibits adenylyl cyclase 6, which in turn leads to a decrease in the cyclic AMP and activated protein kinase levels, thus promoting the transformation of MSC into osteoblasts (Siddappa et al., 2009; Nguyen and Jacobs, 2013).

### 5.2 Bone immune mechanism

Although tissue-engineered bone scaffolds have biocompatibility, the host immune response is an inevitable stage after tissue-engineered bone implantation (Vishwakarma et al., 2016). The initial inflammatory response following biomaterial implantation aids in tissue repair and regeneration; however, persistent inflammation impairs the wound-healing response (Julier et al., 2017).

The material structure and morphology can regulate the bone immune response and promote bone repair (Zheng et al., 2019). When the scaffold was implanted, neutrophils migrated around the scaffold within 24 h and prepared to recruit immune cells by secreting cytokines and releasing neutrophil extracellular traps (NETs). When neutrophils adhere to the surface of the scaffold (extracellular matrix), they are activated to excrete DNA and form neutrophil extracellular traps (NETs) (Schoen et al., 2022; Won et al., 2020) fabricated hierarchically structured “microchannel” 3D printed scaffolds by the 3D printing of a polycaprolactone polymer. Compared to non-microchannel scaffolds made of the same material, the neutrophil-capturing net can be reduced, which is beneficial for tissue repair. Additionally, different levels of extracellular neutrophil capture can be induced by adjusting the template components and diameters during electrospinning, and the capture net can be significantly reduced by increasing the fiber diameter (Fetz et al., 2017).

Macrophages are among the most important immune cells. As early responders after biomaterial implantation, they play a significant role in guiding angiogenesis and tissue remodeling and are closely related to bone remodeling (Guo et al., 2020). Macrophages are usually divided into M1 and M2 phenotypes: M1 macrophages act as pro-inflammatory factors and can release a large number of cytokines such as IL1β, IL8, and TNFα through exosomes, which can cause a series of immune responses (Recalcati et al., 2010; Gao et al., 2016); in contrast to M1, the M2 macrophages mainly release cytokines such as IL10, which inhibit inflammatory responses, promote anabolism such as osteogenesis and angiogenesis, and play a key role in wound healing, tissue repair and other processes (Italiani and Boraschi, 2014; Lee et al., 2019). The surface morphology and microstructure of scaffolds can influence the immune response of the body. The special scaffold structure can reduce macrophages to M1, polarize them to M2, and further regulate angiogenesis and osteogenesis. The most important factors are the particle size (Lebre et al., 2017), porosity (Jordan et al., 2018), and pore size (Chan et al., 2022) of the scaffold structure. Tylek et al. (2020) constructed square porous polycaprolactone fiber scaffolds with different structural shapes using 3D printing technology, with pore sizes ranging from 100 μm to 40 μm. These scaffolds promoted the extension of macrophages and are differentiated into the M2 type, which was most obvious on scaffolds with a pore size of 40 μm. Zheng et al. (2019) regulated the surface structure of the scaffold using near-infrared radiation from a flat surface to a groove-like surface structure, which causes macrophage phenotype changes. Garg et al. (2013) deduced that an increase in the fiber diameter in the electro spun scaffold can promote the transformation of macrophages to M2 macrophages *in vitro*. The 100 nm nanostructure produced on Ti by anodic oxidation is beneficial to M1 macrophages, whereas 30 nm is beneficial to M2 polarization. Cell elongation induces the release of cytokines (IL-4 and IL-13) and polarizes macrophages to the M2-like phenotype, indicating that the cell shape plays a role in the regulation of phenotypic polarization (McWhorter et al., 2013).

### 5.3 Adsorption mechanism of peripheral protein

The influence of the material structure on cells or host reactions is mainly realized by affecting the adsorption behavior of proteins on the surface of materials. When a biomaterial is implanted as a foreign body, its surface is in contact with the extracellular environment, and proteins are usually adsorbed on the surface of the biomaterial earlier than the cells (Puleo and Nanci, 1999). Cells can recognize specific peptide domains in this protein, further regulate their fate, and ultimately affect the biological properties of the scaffolds (Lutolf and Hubbell, 2005). Therefore, increasing protein surface coverage can improve cell adhesion and diffusion (Atif et al., 2022). The structure of the scaffold affects the protein adsorption level. Increasing the number of micropore structures can also increase the interaction between the scaffold and serum proteins, which may be an attractive strategy to promote the osteo-inductivity and osteo-conductivity of scaffolds (Perez and Mestres, 2016). These proteins can then stimulate osteogenic-related cell functions, such as attachment, proliferation, osteogenic differentiation, and biomineralization (Wang et al., 2014). The microporosity and micropore size of the scaffolds have a significant influence on the protein adsorption characteristics. HA and BCP particles with higher and/or more micropores can adsorb more fibrinogen and insulin (Zhu et al., 2010). The rich microporous structure and relatively high body surface area of the scaffold can promote the adsorption of osteogenesis-related proteins, which leads to new bone formation (Campion et al., 2011; Wang J. et al., 2013) studied the effects of calcium phosphate ceramic particles with different structures on protein adsorption using a dynamic protein adsorption device. Under simulated dynamic conditions, the phase composition and microstructure of the CaP ceramics affect their protein adsorption capacity. Among them, spherical hydroxyapatite and biphasic calcium phosphate ceramic particles prepared by spray drying sintering with abundant micropores and high specific surface area have a higher adsorption capacity for serum proteins such as fibronectin and vitronectin, which is beneficial for cell adhesion. Simultaneously, the protein adsorbed on the implanted scaffold can regulate immune activity and play a significant role in macrophage adhesion, activation, and foreign body giant cell formation (Dadsetan et al., 2004).

### 5.4 Cell adhesion mechanism

Cell adhesion is one of the basic life activities of cells, and plays a key role in regulating proliferation, maintaining activity, differentiation and migration. Cell adhesion is related to transmembrane proteins on the cell surface, such as integrin and cadherin (Niessen et al., 2011). These transmembrane proteins can interact with the ECM, directly or indirectly regulate the proliferation of stem cells, and promote cell adhesion and multidirectional differentiation (Abdal Dayem et al., 2018). Some related studies have shown that the micro-nanomorphology of the scaffold surface can affect the adsorption level of cells (Acevedo-Morantes et al., 2012; Li H. et al., 2021). Geng et al. (2020a) simulated a continuous deep pit-like surface structure inside the gill cover of a snail on the surface of a titanium implant to increase the adsorption level of cells. Filova et al. (2015) prepared nanotubes with different diameters by adjusting the voltage of the Ti-6Al-4V alloy. After the co-culture of Saos-2 cells, the adsorption level of the former cells increased significantly compared with that of the cells cultured on glass plates. The surface of this type of metal stent is negatively charged and the surface charge density at the sharp edge is high. Therefore, the surface charge density of small-diameter nanotubes is high, which promotes the adsorption of the fibronectin and vitronectin molecules and proteins with a quadrupolar internal charge distribution, resulting in more effective adhesion and diffusion of osteoblasts to scaffolds and the promotion of osteogenic effects (Kabaso et al., 2011; Gongadze et al., 2013). Different fiber diameters also affect cell adhesion and morphology. When cells contact coarse fibers, they tend to adhere to the surface of the coarse fibers as a whole and fill pores in a circular manner; When coming into contact with fine fibers, the cells tend to wrap fine fibers at one end, thus forming a “bypass”. Consequently, the cells exhibited an obvious directional growth trend on a specific arrangement of thick and thin fibers (Xie et al., 2019). Moreover, the richer the hierarchical structure of the materials, the richer the adsorption effect. Wang et al. prepared a TiO_2_ nanotube structure based on a micron trabecular bone structure by anodic oxidation and formed a micro-nano gradient coexistence bionic structure. Compared to pure titanium and micron-trabecular bone groups with lower structural levels, it was deduced that a rich hierarchical structure can effectively promote the adhesion, proliferation, and osteogenic differentiation of BMSCs (Jang et al., 2017). For example, adding nanoscale structures on the surface of the scaffold using a laser can promote the adhesion function of BMSCs and promote osteogenesis (Šugár et al., 2021). Bone progenitor cell differentiation can point to the osteoblast phenotype by reducing the size of the nano-morphology to 10 nm (Dalby et al., 2006b).

## 6 Influence of manufacturing process on scaffold structure

The fabrication process affects the structure of the scaffold. Generally, to prepare a specific scaffold structure, it is necessary to use a specific preparation method. Presently, the most common methods for preparing pore scaffolds are freeze-drying (Brougham et al., 2017), electrospinning (Yan et al., 2020), and gas foaming (Chen et al., 2021); however, these methods cannot effectively control the pore structure. With the update of 3D printing technology, new technologies such as digital light projection printing can quickly manufacture composites with complex pore structures, adjust the pore structure parameters (Zhang et al., 2022a; Song et al., 2022), and accurately control the shape of scaffolding. Therefore, geometric structures with different shapes can be accurately manufactured by computer aided design (CAD) (Zopf et al., 2015), such as complex geometric objects or artificial organ frames. For the topological structure of the scaffold surface, nano-coating is often used to cover the scaffold surface by hydrothermal deposition, or the scaffold surface is patterned directly by laser micromachining (Aguilar et al., 2005; Geng et al., 2020) prepared the texture topology of the operculum of a river snail on a Ti surface using electrochemical corrosion and anodic oxidation. Strontium-doped apatite was then deposited on the surface by hydrothermal deposition. Moreover, 3D printing technology can be easily processed for microchannel structures with a simple structure. Microchannels with complex structures can be constructed using the sacrificial template method (Jeon et al., 2023; Saggiomo and Velders, 2015)proposed a simple two-step acrylonitrile butadiene styrene (ABS) scaffold removal method that can be used to realize 3D multilayer complex micron channels in a single block of polydimethylsiloxane.

## 7 Outlook, perspective, and conclusion

The process of the osteogenic differentiation of mesenchymal stem cells is influenced by the external matrix. In natural bone, cells grow in an external matrix with a hierarchical structure, after which, the scaffold is implanted into the body and the extracellular environment is exposed in the scaffold. Therefore, by imitating the natural bone structure, optimizing the scaffold structure can regulate cell growth and differentiation and provide a suitable external environment for cells. Scaffolds with different structures have different functions, such as macropores and microchannels, which are beneficial for blood vessel growth; a concave surface is beneficial for bone formation and micropores are beneficial for cell adsorption. These functions can affect the characteristics of the scaffold, and the scaffold can be used to the maximum extent using a reasonable design. Technological innovations in preparation methods such as photo-curing 3D printing and the sacrificial template method, or through scaffold hierarchical structure innovations such as bionic technology to improve the scaffold structure, create new possibilities for new micro-nano bionic scaffolds and the development of bone tissue engineering. Moreover, the osteogenic mechanism of the scaffold structures remains unclear. It mainly is in the classical pathway but lacks the interaction between scaffolds and signal molecules. In the future, through transcriptome analysis, single-cell sequencing and other technologies can be used to deeply explore the principle of the structural influence on osteogenesis.
